# Larger colony sizes favour greater division of labour between queens and workers in ants

**DOI:** 10.1093/evolut/qpaf250

**Published:** 2026-02-18

**Authors:** Juliet Turner, Louis Bell-Roberts, James McCulloch, Matilda Brindle, Rosa Bonifacii, Stuart West

**Affiliations:** 1 Department of Biology, University of Oxford, Oxford, United Kingdom; 2 Wellcome Sanger Institute, Hinxton, Cambridgeshire, United Kingdom; 3 Department of Zoology, University of Cambridge, Cambridge, United Kingdom

**Keywords:** division of labour, worker sterility, ants, the size-complexity hypothesis

## Abstract

Explaining variation in the extent of division of labour remains a major problem for our understanding of how complex life evolved. Ants show remarkable variation in their extent of reproductive division of labour, from workers who can reproduce sexually and are approximately the same size as queens, to workers that are completely sterile and 300x smaller than their queens. Examining data from 546 species of ant, we found that: (i) the ancestral ant worker likely had full reproductive potential, though was effectively sterile in the presence of a queen; (ii) the loss of worker reproductive potential generally followed a sequential step-by-step process, via reduced capacity for sexual reproduction, then the production of males only, and finally complete sterility; (iii) the independent evolution of complete sterility has occurred approximately 17 times, with only 42% of ant species having sterile workers; (iv) reproductive size dimorphism has increased to higher levels around 9 times. Exploring potential causality, we found support for the size-complexity hypothesis, that increased colony size has favoured increased division of labour between queens and workers, examining both queen-worker size dimorphism and the loss of reproductive capacity in workers.

## Introduction

Division of labour has played a pivotal role in driving evolutionary transitions to more complex life (Boomsma, 2022; Bourke, 2011; West et al., 2015; Szathmary & Maynard-Smith, 1995). A major evolutionary transition in individuality is when individuals join to form a new higher-level, and more complex entity. For example, genes formed genomes, cells formed multicellular organisms, and animals formed superorganismal societies. Division of labour is fundamental to these transitions as it leads to individuals becoming reliant upon each other for reproduction (mutual dependence). Consequently, a major aim is to understand where division of labour has, or has not, evolved.

**Figure 1:**
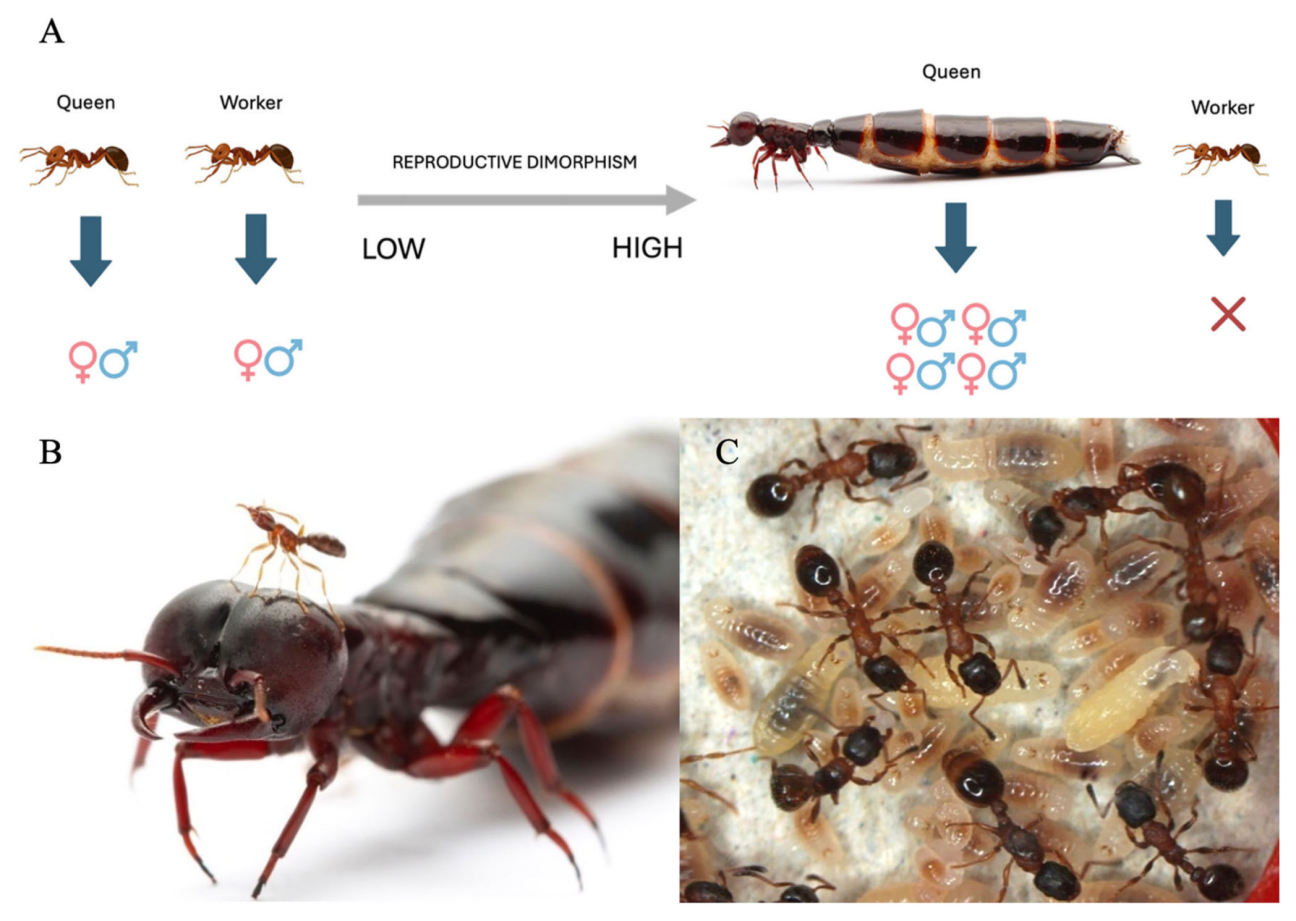
Reproductive dimorphism varies across species. (A) Reproductive dimorphism in ants can arise via size dimorphism and differences in reproductive capacity. (B) *Dorylus* worker perches on the head of a queen, illustrating extreme reproductive dimorphism. (C) *Leptothorax* queens and workers provide an example of minimal reproductive dimorphism. (Photos: A & B: Daniel Kronauer, C: Walter & Heinze, 2015).

Ants show considerable variation in the extent of reproductive division of labour between the queens (reproductives) and their workers (helpers) (Hölldobler & Wilson, 1990; Matte & LeBouef, 2025; Vizueta et al., 2025). The workers of some species are born with the potential to mate and produce diploid offspring, while in other species they are completely sterile. Similarly, some species have workers and queens of approximately equal size, while others have queens more than 300 times larger than the workers (Figure 1). How can we explain this variation? How often has the extreme reproductive division of labour represented by sterility evolved in ants?

The size-complexity hypothesis provides a potential explanation for variation in the extent of reproductive division of labour across ants. It predicts that in lineages that have undergone a major transition to a higher-level organism, larger numbers of lower-level subunits select for increased division of labour (Bell & Mooers, 1997; Bell-Roberts et al., 2024; Bonner, 2004; Boomsma, 2022; Bourke, 1999; Bourke, 2011; Oster & Wilson, 1978;). Support for the size-complexity hypothesis has been provided through evidence that multicellular organisms with larger numbers of cells have more cell types (Bell & Mooers, 1997; Bonner, 2004; Fisher et al., 2013). Consistent with this, ant species with larger colonies have a greater queen-worker dimorphism (Fjerdingstad & Crozier, 2006; Kramer & Schaible, 2013; Bourke, 2011; Ferguson-Gow et al., 2014; Matte & LeBoeuf, 2025; Vizueta et al., 2025). Larger colony sizes have been hypothesised to favour increased size dimorphism between workers and queens because of reduced reproductive replacement opportunities for workers and increased fecundity required for queens (Bourke, 1999). However, the possible influence of colony size on the other key aspect of reproductive division of labour—worker sterility—has not been tested.

The possible influence of colony size on reproductive division of labour remains unclear. First, as discussed above, the influence of colony size on the evolution of worker sterility has never been tested. Second, the observed correlations between colony size and reproductive size dimorphism are correlational and open to multiple interpretations (Cornwallis & Griffin, 2024). While larger colony sizes could have favoured greater size dimorphism, the reverse causal relationship is also possible, that greater size dimorphism favoured larger colony sizes. For example, increased size dimorphism could have increased colony efficiency, or smaller workers could lead to larger colonies. Alternatively, greater size dimorphism could have been favoured by another factor that is correlated with colony size. For example, the number of males a queen mates with or the number of reproductive queens per colony, could influence the evolution of both reproductive division of labour and colony size (Cooper & West, 2018). Matte & LeBoeuf (2025) examined the evolution of queen-worker size dimorphism but could not determine whether larger colony sizes favoured increased size dimorphism or the reverse. Ultimately, distinguishing between these competing explanations requires phylogenetic analyses that examine the order of evolutionary change and test for the most likely causal explanation.

**Figure 2:**
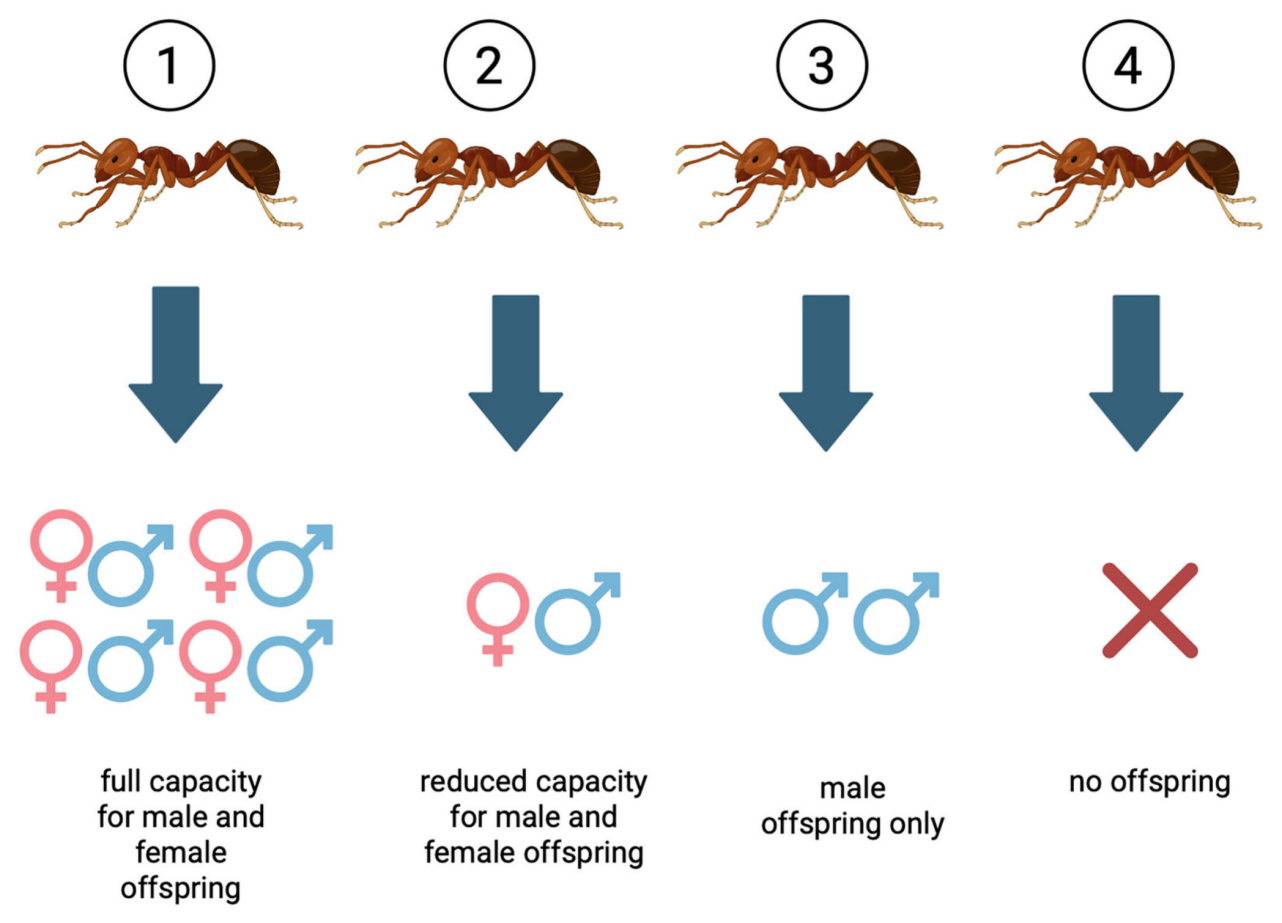
Variation in physical reproductive capacity of ant workers. Ant species were assigned to one of four categories of worker physical reproductive potential. 1 = full capacity to produce male and female offspring, 2 = reduced capacity to produce male and female offspring, 3 = capacity to produce male offspring only, 4 = no capacity to produce offspring. The numbers indicate degree of sterility but do not imply any particular evolutionary order, i.e., they are categorical, not ordinal (inspired by Khila & Abouheif, 2010, and similar to Gotoh et al., 2016).

We investigated the evolution of reproductive division of labour across 543 species of ants, examining both worker sterility and queen-worker size dimorphism. For worker sterility, we considered both the “reproductive potential” of workers, in terms of what they are physically capable of at birth (Figure 2), and the “realised reproduction” of workers, in terms of their level of reproduction within a normally functioning colony. Our aims were to: (1) determine how often extreme reproductive division of labour has evolved in ants, as represented by loss of worker reproductive potential or extreme queen-worker size dimorphism; (2) use phylogenetic comparative analyses designed to test causal hypotheses about how the evolution of these two different aspects of reproductive division of labour have been influenced by three potential explanatory variables—colony size, the number of males that queens mate with (polyandry) and the number of queens (polygyny) (Fjerdingstad & Crozier, 2006; Frumhoff & Ward, 1992; Oster & Wilson, 1978).

## Materials and methods

### Measuring reproductive division of labour

We measured reproductive division of labour in two ways: the ability of workers to reproduce and queen-worker head size dimorphism.

#### Colony size and queen mating frequency

We used information on colony size and queen mating frequency from the dataset published previously in Bell-Roberts et al. (2024). Our calculation of colony sizes used the total number of workers across all nests in a colony, so our colony sizes for polydomous species will be the total number of workers in their colony, not just in a single nest.

#### Measuring worker reproduction

We used a Web of Science search to gather data on worker reproduction for all species where we had colony size or queen mating frequency data. We used the search terms:

$$\left((\left(\;{\text{ovar}}^{*}\right)\;\text{OR}\;\;({\text{steril}}^{*}\right)\;\text{OR}\;(\text{reproductive}\;-\;\text{constraint}*)\;\text{OR}\;(\text{ants})\;\text{AND}\;(\text{species}\;\text{name})))$$

We assigned each species to one of four categories of worker physical reproductive potential: 1 = full capacity to produce male and female offspring, 2 = reduced capacity to produce male and female offspring, 3 = capacity to produce male offspring only, 4 = no capacity to produce offspring (Figure 2).

The concepts of “full” and “reduced” reproduction are defined relative to a more reproductive (queen) caste. In species where workers can sexually reproduce but a queen caste with greater reproduction is present, the workers would be categorised as having “reduced” capacity. In contrast, in species with no caste with greater reproductive capacity, sexual workers could be considered to have “full” capacity.

We categorised worker reproduction in two ways. First, we considered the “reproductive potential” of workers, in terms of their physical potential to reproduce at birth. Second, we considered the “realised reproduction” of workers, in terms of how much they reproduce in normal colonies with queens present. The reproductive potential and realised reproduction of workers can differ for several reasons, including behavioural interactions with or policing by other workers or queens (Bourke, 1999).

#### Measuring queen-worker head size dimorphism

We used head size to estimate the difference in size between queens and workers as it is the most reported measure of queen and worker size in the literature and correlates with body size (Ferguson-Gow et al., 2014; Hölldobler & Wilson, 1990; Kaspari, 1993; Vaino et al., 2004;; Matte & LeBoeuf, 2025). We used the AntWeb online database (antweb.org) to find scaled images of all the species where we had previously found colony size, queen mating frequency, and queen number data (Bell-Roberts et al., 2024). Usually, several images of each specimen were included in the website’s species pages to aid identification. This included front-view images of the ants’ heads, which we downloaded and measured at the widest point excluding the eyes using the image-processing software ImageJ. The number of workers measured per species ranged from 1–37, with a mode of 5. The number of queens measured per species ranged from 1–10, with a mode of 1.

Some species have multiple worker castes, which can vary in size. We are interested in the evolution of extreme specialisation, so we measured maximum size dimorphism within a species, by using the minimum worker head width (i.e., smallest individual worker). Specifically, we measured reproductive size dimorphism as:

Reproductivesizedimorphism=meanqueenheadwidth/minimumworkerheadwidth

A value of reproductive dimorphism equal to 1.0 implies that workers and queens are the same size, while values > 1.0 imply that queens are larger than workers. We were able to compile head size information for a total of 1,062 workers and 132 queens.

### Analyses

We began by testing alternative models of state evolution to build a picture of the evolutionary history of worker reproductive potential. We then used phylogenetic regressions to test for correlations between the variables that we measured. Finally, we used three methods to test hypotheses about the likely causality of these correlations: (i) phylogenetic path analysis, (ii) transition rate analysis between pairs of traits, and (iii) ancestral state reconstruction. We used different methods to both examine different but closely related questions, and to test the robustness of our results. We always analysed with the maximum number of species, where data for all the variables being considered was available. Consequently, the number of species varied across analyses because of variation in the data availability. We retained continuous variables as continuous, except when the form of analysis required that we binarize them.

Before running the analyses, we filtered out any species without the required data and any species known to be supercolonial, socially parasitic, clonal, or hybrids (Bell-Roberts et al., 2024; Helanterä, 2022). These species have very different life histories, meaning that we would need to make different predictions, and our variables cannot always be measured in the same way. We categorised all species by whether the reproductive state of their workers was explicitly described (high confidence subset) or inferred from congeners in cases of no known intrageneric variation (all species subset) and repeated all analyses with these two subsets. Each analysis used the largest set of species that had data for both the predictor and response, so sample sizes vary across analyses.

As trait similarity may be the result of shared ancestry, all our methods controlled for phylogeny (Dewar et al., 2025). We used different numbers of phylogenetic trees depending on the method employed. For Bayesian regressions and ancestral state reconstructions, we incorporated phylogenetic uncertainty directly by running models across 400 posterior trees. Phylogenetic path analysis, however, relies on frequentist statistics and does not readily allow for integration across a posterior sample of trees. For this reason, we used four maximum clade credibility (MCC) trees generated from different tree-building methods. This provided an alternative way of testing whether our results were consistent across plausible phylogenetic relationships.

#### Order of state evolution

The variation that we see in ant worker reproductive potential could have arisen in different ways. It is possible that states 1 (full sexual capacity) and 4 (no offspring) represent extreme ends of a continuum, and that to reach either extreme, species must pass through the intermediate states as steps on a ladder of increasing or decreasing social complexity. However, an alternative possibility is that the four states could be non-ordinal categories, where one state can transition directly into any other (Supplementary Figure S1).

To test which model has stronger support, we used MultiState in BayesTraits (Pagel, 1994). The free model was run with the following priors: HyperPriorAll exp 0 0.1, scaletrees 0.001, burn-in 3,000,000, iterations 30,000,000, stones 100 10000. For the restricted (sequential) model, we ran with the same priors except that we restricted non-sequential transitions to zero. We also tested two intermediate models in which non-sequential transitions could occur but were rarer than the sequential transitions. We compared the models in a pairwise manner (simpler model vs complex model; Supp Info Table 1).

### Phylogenetic correlations

We tested whether colony size, queen mating frequency (estimated number of mates weighted by the proportion of offspring sired by each male), or number of queens per colony were correlated with the worker reproductive potential or queen-worker dimorphism of ant species in a series of pairwise simple phylogenetic regressions. Colony size was log_10_ transformed and colony size, mating frequency, queen number, and queen-worker dimorphism were analysed as continuous variables. Worker reproductive potential was analysed as categorical, divided between the four categories outlined in Figure 2 (1 = full capacity for male and female offspring, 2 = reduced capacity for male and female off-spring, 3 = male offspring only, 4 = no offspring). Ants have a haplodiploid sex determination system where male offspring are haploid and hatch from unfertilised eggs, while female offspring are diploid and hatch from fertilised eggs. There are rare cases of species where diploid female off-spring can be produced clonally but these were among species filtered out prior to our analyses. Species in categories 1 & 2 (capacity for male and female offspring) can therefore be considered the species with worker mating, while those in categories 3 & 4 (male offspring only & no offspring) have workers unable to mate.

We performed all regressions in R v 4.2.3. We controlled for phylogenetic non-independence by running Bayesian phylogenetic generalised linear mixed models (BP-MMs) with the R package “*MCMCglmm*” version 2.34 (Cornwallis & Griffin, 2024; Hadfield, 2010). We used the package “mulTree” version 1.3.7 to repeat the analyses over all the possible ant phylogenetic trees produced by Economo et al. (2018). Economo et al. produced 100 posterior trees with each of four methods, giving a total of 400 possible trees. We repeated each regression analysis over all 400 trees to examine the effects of phylogenetic uncertainty. At present, only genus-level phylogenies exist for ants and species topology is largely unknown. Therefore, we want to know whether this uncertainty can change our results.

We ran all models for at least 11,000,000 iterations with a burn-in of 1,000,000 and a thinning interval of 5,000. We used the “*coda*” package version 0.19–4.1 to calculate the degree of autocorrelation between successive iterations in each chain. We fitted each model independently two times and used Gelman and Rubin’s convergence test to compare within- and between-chain variance (Gelman & Rubin, 1992; Plummer et al., 2006). We encountered convergence issues with the models when setting worker reproductive potential as the response variable (*categorical* family distribution), and so to test the robustness of results, we also ran the models with predictor and responses swapped. It is possible that the relationship effects could be in either direction. In those cases, we applied a *gaussian* family distribution to the colony size regression and *exponential* to queen number and mating frequency.

#### Phylogenetic path analysis

We used phylogenetic path analyses (“*phylopath*” package version 1.1.3; Von Hardenberg & Gonzalez-Voyer, 2012) to evaluate alternative causal models for the evolution of queen-worker dimorphism and worker reproductive potential, including paths of direct and indirect influence among the variables. Path analysis compares alternative causal models and disentangles direct and indirect effects between variables. The models we tested were based on the results of our correlational analyses (Supplementary Figures S5 & S6). This technique uses frequentist statistics, meaning we cannot combine posterior samples run over 400 trees, and so we instead ran our analyses with four Maximum Clade Credibility (MCC) consensus trees and compared the results.

All variables were analysed as continuous or, in the case of worker reproductive potential, categorial.

#### Correlated trait evolution and transition rate analysis

We used *BayesTraits* V4.0 (Meade & Pagel, 2023) with Discrete MCMC estimation to test for correlated evolution between (i) worker reproductive potential and colony size, (ii) worker reproductive potential and queen number, (iii) queen-worker size dimorphism and colony size, and (iv) queen-worker size dimorphism and queen mating frequency. We compared the fit of a dependent model of evolution (in which the state of one variable is dependent on the state of the other), with that of an independent model in which traits evolve separately.

*BayesTraits* requires both traits to be modelled as binary variables: Worker reproductive potential (originally four categories) became (i) sex/no sex (categories 1, 2 vs 3, 4) and (ii) non-sterile/sterile (categories 1, 2, 3 vs 4). Queen-worker size dimorphism (initially a continuous variable) became low size dimorphism vs high size dimorphism, defined by whether values were above or below the median, excluding a 5% buffer zone. Colony size was transformed into a binary variable using the threshold of whether it fell above or below the median excluding a 10% buffer zone. We had more species with colony size data so could afford to discard more of them with a larger buffer zone. Queen number and queen mating frequency both used the threshold of above or below two. Having two or more queens per colony (polygyny) means that workers are not full siblings and therefore maximal relatedness cannot be achieved. Consequently, different predictions have been made about the expected reproductive behaviours of workers in single queen (monogynous) vs multiple queen (polygynous) colonies (Bourke, 2001). Similarly, two or more matings for queens (polyandry) reduces relatedness among workers, resulting in an average worker relatedness that is halfway between the theoretical maximum (0.75) and minimum (0.25) for single-queen colonies. Additional matings beyond two have a progressively smaller impact on relatedness, and mating with two males marks the point at which worker policing is favoured (Bell-Roberts et al., 2024; Ratnieks, 1988).

We scaled the tree, so the branches had a mean length of 0.1 (as recommended in *BayesTraits* V4.0 manual), enabling the algorithm to better explore parameter space and preventing rates from becoming very small or difficult to estimate. Scaling the branches does not affect results, since the parameter space of the transition rates is scaled by the same constant (Meade & Pagel, 2023). All models employed reversible-jump Markov chain Monte Carlo (rjM-CMC) methods and an exponential prior, with a mean drawn from a uniform hyperprior ranging from 0 to 0.1. All models were run for 1 billion iterations with a burn-in of 1,000,000 iterations, sampling every 10,000 iterations. Analyses were repeated three times and the median log like-lihood value was reported. We also repeated across all 400 posterior trees from Economo et al., 2018.

Trace plots were visually examined in *Tracer* V1.7.2 (Rambaut et al., (2018)) to check chain mixing and convergence. Parameter acceptance rate was examined via the schedule file. All effective sample sizes were at least > 3,000. Each model was run three times to check for consistency, and that with the median log marginal likelihood was chosen. Model support was evaluated using Bayes Factors (BFs). Natural log marginal likelihoods of both the independent and dependent models were estimated using a stepping stone sampler (Xie et al., 2011), set with 100 stones, running for 10,000 iterations and the default parameters of *α* = 0.4 and *β* = 1 (Xie et al., 2011). BFs were interpreted following Kass & Raftery (1995): 0–2, minimal support; 2–6, positive support; 6–10, strong support; greater than 10, very strong support.

#### Ancestral state reconstructions

To better understand the order of trait evolution and the relationship between colony size and our two measures of reproductive dimorphism, we carried out ancestral state reconstructions and analyses at transition points (Cornwallis et al., 2017; Hadfield, 2010). We examined: (i) whether colony size was higher at the ancestral nodes of lineages that had transitions to a state of high dimorphism versus those that didn’t; and (ii) whether colony size was higher preceding or following transitions to high dimorphism. If colony size is higher in lineages that undergo the transition versus those that don’t (part i), this would suggest that large colony size facilitated the transitions to high dimorphism. If colony size is higher after the transition to high dimorphism than it was before the transition, it would suggest that transitions in dimorphism facilitated the evolution of larger colonies.

For these analyses we transformed our measures of reproductive division of labour into binary variables (as described in *Correlated Trait Evolution*) but kept colony size, queen number, and queen mating frequency continuous. First, we reconstructed the ancestral states of worker sterility (sterile/non-sterile), worker sex (sex/no sex), and queen-worker size dimorphism (low/high) to find where these transitions occurred. We ran the analysis with all 400 posterior trees (Economo et al., 2018) with the “*corHMM*” package, version 2.8 (Beaulieu et al., 2013) and ran models with different rate categories (1–3 rates) and models of evolution (ER *equal rates* and ARD *all-rates-different*). We used AIC values to identify the best model. We then used Bayesian Phylogenetic Mixed Models (*BPMMs*; Hadfield, 2010) to examine (i) whether colony size was higher at the ancestral nodes of lineages that had transitions to a state of high dimorphism versus those that didn’t and (ii) whether colony size was higher preceding or following transitions to high dimorphism.

## Results

### Worker reproductive potential

**Figure 3:**
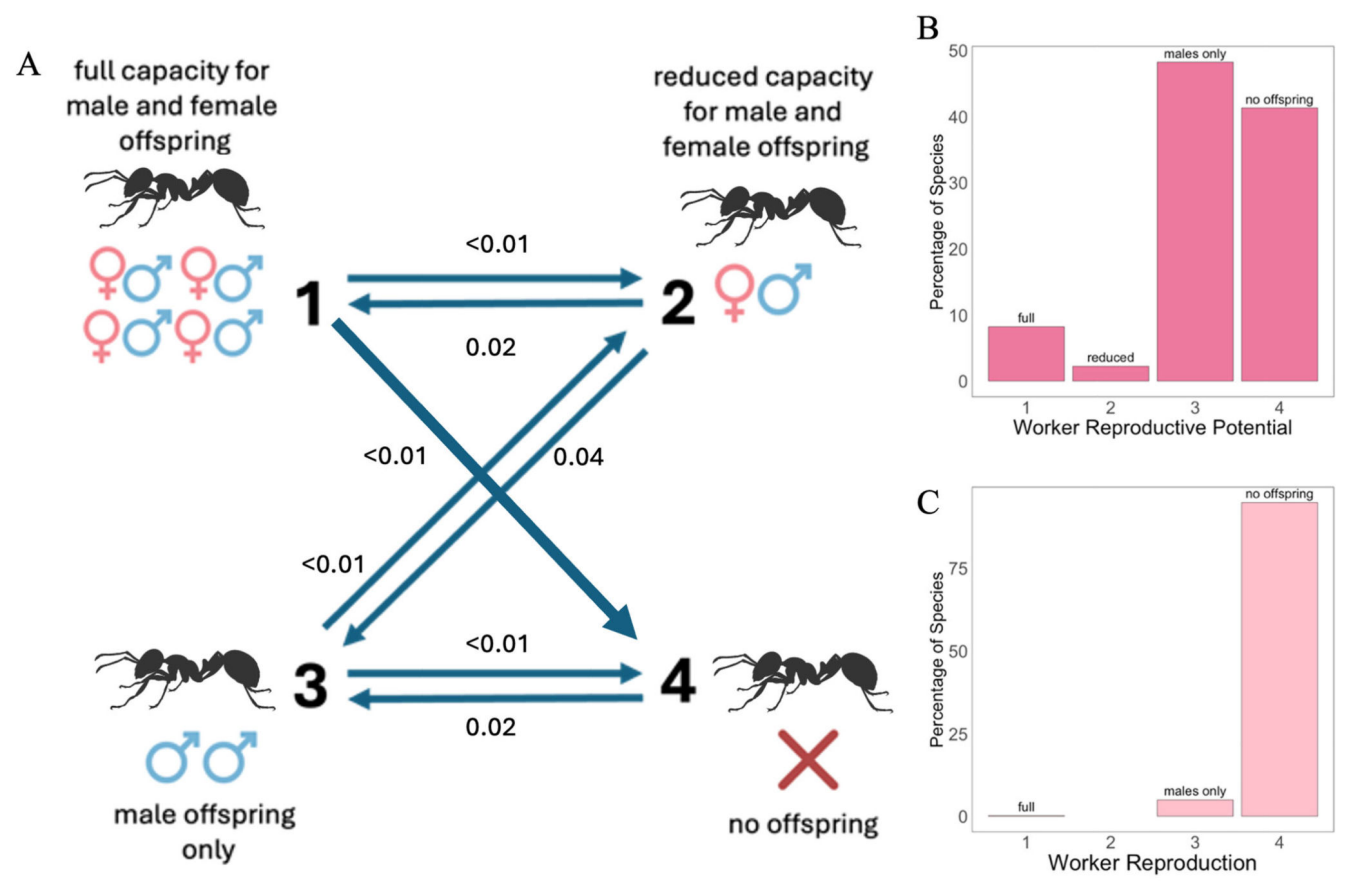
Worker reproductive potential. (A) Support for the mostly sequential model of trait evolution. Values are mean transition rates between states of worker reproductive potential at birth. We found strongest support for the model of evolution where ant workers lost or gained reproductive potential in a mostly sequential manner, except for one transition (full capacity → no offspring) where complete loss of potential occurred in one step. (B) Distribution of worker reproductive potential across 535 species of ant (their physical capacity) and (C) Distribution of realised worker reproduction across 522 species (the actual reproduction that workers do in a normally-functioning colony; not counting gamergate queens as workers)—not used in correlational or causal analyses owing to lack of variation.

We categorised 546 species of ant by the reproductive potential of their workers. We found that 58% of ant species have workers with some potential for reproduction (Figure 3B). The majority of those are only able to produce male offspring (47% of all species). However, 11% of species have workers able to produce both male and female off-spring, comprised of 10% sexually reproducing species, and 1% species clonally reproducing. We filtered out the clonal species and further divided the species where workers could reproduce sexually by whether they have “full” or “reduced” sexual capacity in relation to the queens (8 & 12% of the total respectively). The remaining 42% of the species had workers that were completely sterile.

We found a very different distribution of worker realised reproduction when compared to the distribution of worker physical capacity. Almost all species for which data were available (95%) show no worker offspring production in normal functioning colonies, with only 5% of species producing males (Figure 3C, n _species_ = 522). This did not provide enough variation for correlational or causal analyses, and so we focused our further analyses on worker physical reproductive potential.

### Order of state evolution

We found strongest support for a model of evolution where transitions in worker reproductive potential occur predominantly in a stepwise sequential manner, except for one non-sequential transition (Figure 3A). This suggests that ant workers usually lost their reproductive potential in ordered steps: first reduced capacity for sexual reproduction, then the production of males only and then complete sterility (1 → 2 → 3 → 4). The exception was one non sequential transition type, where states of full sexual capacity transitioned to no offspring (1 → 4), which can either be interpreted as a loss of all reproductive potential in one step, or as step-wise losses that happened so rapidly that the intermediate steps could not be detected. We reached this conclusion by testing different models against each other in each pairwise comparison (Supp Info Table 1). Note that this result does not mean that the realised worker reproduction gradually decreased.

### Ancestral state reconstruction of worker reproduction

The ancestral ant worker most likely had full reproductive potential, but was effectively sterile in the presence of a queen (most likely root state: = category 1 when looking at reproductive potential, category 4 when looking at realised reproduction).

The credible intervals for all states of reproductive potential include values close to zero, suggesting that while “full potential” has the highest probability, there remains considerable uncertainty (Supplementary Figure S4; full reproductive potential = 0.59, CIs = <0.01–0.97, reduced reproductive potential = 0.11, CIs = <0.01–0.63, male offspring only = 0.11, CIs = <0.01–0.45, completely sterile = 0.13, CIs = < 0.01–0.71, n _species_ = 389; Supplementary Figure S7). However, when examining realised reproduction, the root state of “no offspring” was far more likely than the others (full reproductive potential < 0.01, CIs = <0.01–0.01, male offspring only 0.03, CIs = <0.01–0.07, no offspring = 0.78, CIs = 0.50–0.99, n _species_ = 368; Supplementary Figure S8).

**Figure 4:**
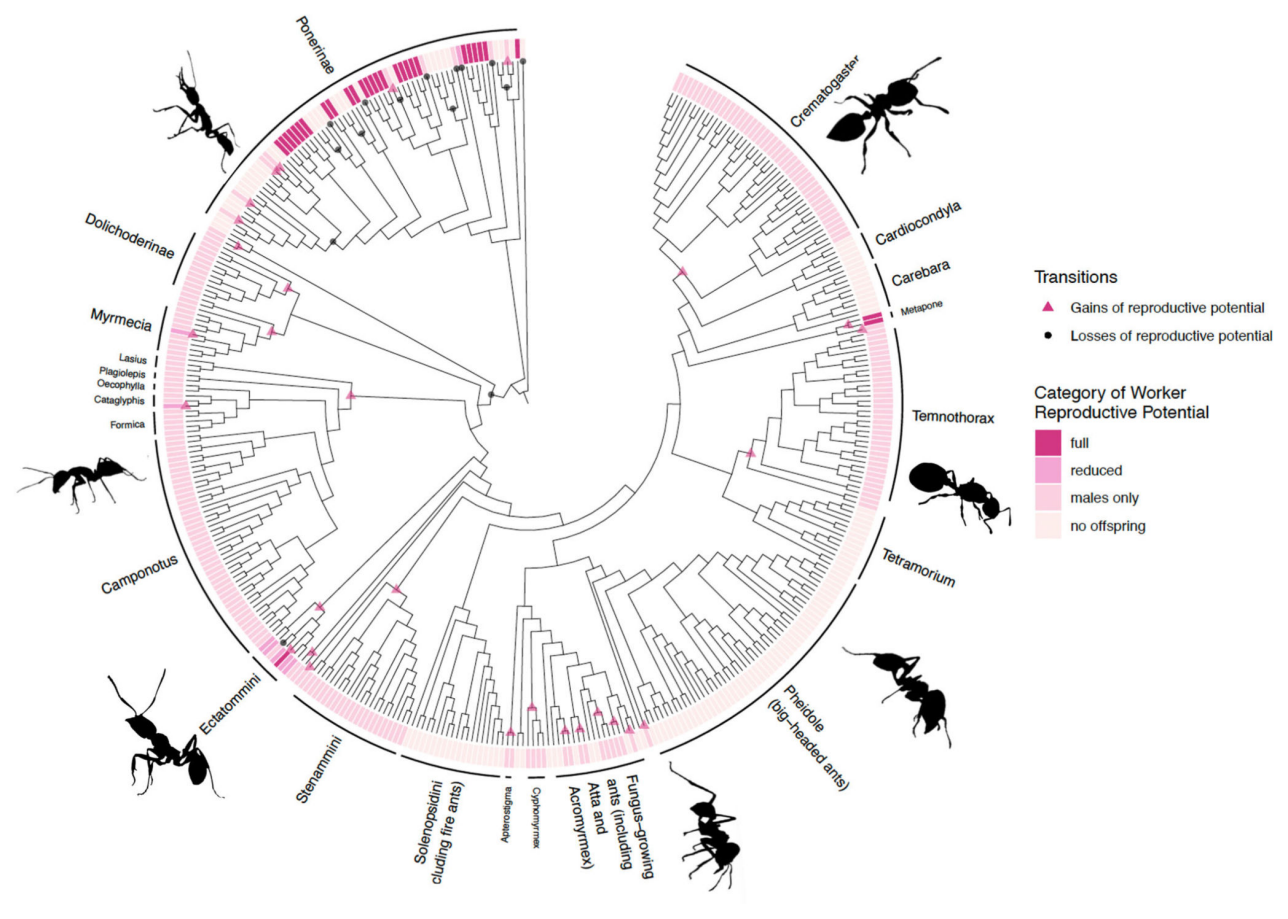
Ancestral state reconstruction of worker reproductive potential. We reconstructed worker reproductive potential as a four-level categorical variable using the all-rates-different model with two rate categories. Plot shows transitions of only one tree, but we repeated across 400 possible phylogenies to calculate mean transition numbers (n _species_ = 389).

We estimated that there have been a mean of 5 gains and 16 losses of the capacity to reproduce sexually in ants (transitions between full or reduced sexual capacity versus male production only or no offspring; categories 1 & 2 ←→ 3 & 4); and a mean of 17 gains and 20 losses of complete worker sterility (transitions between full or reduced sexual capacity or male production versus no offspring/complete sterility; categories 1, 2, & 3 ←→ 4) (Figure 4). The evolution of decreased reproductive potential in workers was associated with larger colony sizes, but this association was not significant (Supplementary Figure S3).

### Correlational analyses

We found that ant species with less worker reproductive potential have larger colonies and more queens, but showed no correlation with queen mating frequency (BPMM: colony size and worker reproductive potential: pMCMC = 0.015, CI = 0.07–0.56, n_species_ = 221; queen number and worker reproductive potential: pMCMC = 0.04, CI = 0.02–0.81, n_species_ = 98; queen mating frequency and worker reproductive potential: pMCMC = 0.950, CI = -0.66–0.68, n _species_ = 60). Queen-worker size dimorphism was not a significant predictor of worker reproductive potential (pM-CMC = 0.266, CI = -0.2765–1.0043, n_species_ = 32).

### Phylogenetic path analyses

**Figure 5:**
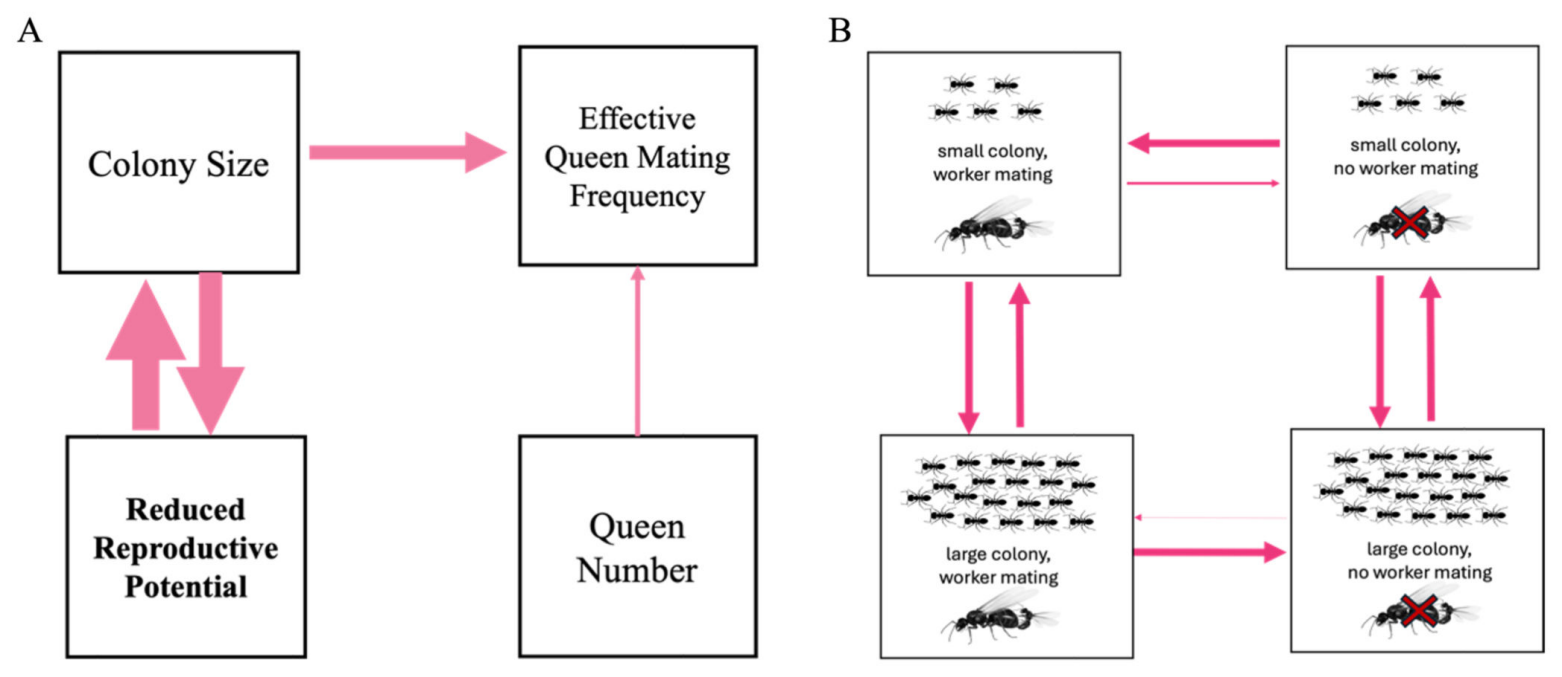
The best-supported causal models identified by phylogenetic path analysis and correlated evolution tests. (A) Path analysis. Best-supported causal model identified by phylogenetic path analysis: Reduced worker reproductive potential favours larger colony sizes, and larger colony sizes favour reduction of worker reproductive potential. We analysed reproductive potential as four categories (categories 1, 2, 3, 4; Figure 2). All other variables are continuous. We display the results of our highest confidence data (n_species_ = 44) and the FBD Crown MCC consensus tree, but we repeated the analyses for three others which can be found in the Supplementary Data. Arrows indicate the direction of the relationship between variables, with thicker lines indicating larger path coefficients. Dashed arrows would indicate a negative relationship, while unbroken lines indicate positive, (B) Correlated evolution. The best-supported causal models identified by correlated evolution tests: Larger colony sizes favour the loss of worker mating. Workers rarely lose sexual capacity in small colonies and rarely gain sexual capacity mating in large colonies. Values represent mean transition rates, with thicker arrows indicating higher rates. Here we display the results of our highest confidence data (n_species_ = 124). Both reproductive potential and colony size are binarized, with reproductive potential analysed as (sex/no sex) and colony size as (below/above median colony size 214).

We found consistent support for the size-complexity hypothesis, but with a bidirectional relationship between colony size and worker reproductive potential. This suggests that larger colony sizes favoured the loss of reproductive potential, and the loss of reproductive potential favoured larger colony sizes (Figure 5A, n _species_ = 44). This conclusion was robust across analyses with different levels of certainty (whether worker reproductive potential was inferred or described), different phylogenetic trees, and the inclusion of supercolonial species (Supplementary Data tabs 3 & 4; Supplementary Data Table 9). In contrast, we did not find evidence of an effect of queen number on loss of worker reproductive potential. The results were similar when analysing loss of worker reproductive potential as binary, dividing species by which have workers that can mate and those with no worker mating (Supplementary Figure S4).

### Correlated evolution

We again found support for the size-complexity hypothesis with larger colony sizes having favoured reduced worker reproductive potential (Figure 5B, n _species_ = 124). Correlated evolution analyses require worker reproductive potential to be analysed as a binary variable. This involved comparing species where the workers reproduced sexually (producing males and females, categories 1 & 2) or not (producing just males or no offspring, categories 3 & 4). We found evidence that larger colony sizes favoured the loss of worker sexual reproduction. This involved comparing species where the workers reproduced sexually (producing males and females, categories 1 & 2) or not (producing just males or no off-spring, categories 3 & 4). The strength of the correlation between colony size and worker sexual reproduction was positive to strong (BF 2.6–5.6; Meade & Pagel, 2023). We found that ant workers more often lose the ability to reproduce sexually in large colonies, but rarely in small colonies (and the opposite with the gain of sexual reproduction). This was robust to different classifications of colony size and whether worker reproductive potential was explicitly described or inferred from genus-level information (Supplementary Data Table 1).

In contrast, we found no evidence that loss of worker sexual reproduction influences colony size (Figure 5B). We also did not find evidence for colony size influencing a different aspect of reproductive potential (complete sterility) or for correlated evolution between queen number and worker reproductive potential. As an alternative analysis, we binarized reproductive potential by dividing between species where the workers could reproduce (categories 1–3) or were completely sterile (category 4). We found no evidence for correlated evolution between colony size and complete worker sterility, suggesting that the capacity for male production is not dependent on the size of the colony (median threshold BF = -5.7, mean threshold BF = -1.07). We also found no evidence for correlated evolution between queen number and worker sex or worker sterility, suggesting that the loss or gain of worker reproductive potential is not dependent on the number of queens in the colony (worker sex and queen number BF = -1.1, worker sterility and queen number BF = -3.8; Supplementary Data Table 1).

#### Queen-worker size dimorphism

We measured worker head size of 153 species, and queen head size of 132 species, and found that worker head size ranged from 0.29mm to 5.59mm, while queen head size ranged from 0.25mm to 5.26mm. After filtering, we have an overlap of 76 species for which we could calculate queen-worker dimorphism. We found that the reproductive size dimorphism, as measured by mean queen head width/minimum worker head width, varied from 0.84 (Harvester Ant *Veromessor pergandei*) to 7.03 (Driver Ant *Dorylus nigricans*; Figure 1). Assuming that overall size is proportional to width^3^ this means that means that the greatest dimorphism involves queens being approximately 350 times the overall size (volume) of workers.

### Correlations

When looking at queen-worker size dimorphism, we found that ant species with greater queen-worker size dimorphism have larger colonies and higher effective mating frequency, while there was no significant correlation with the number of queens per colony (BPMMs: colony size pMCMC = <5e-04, CI = 0.03–0.08, n_species_ = 54; queen mating frequency pMCMC = <5e-04, CI = 0.06–0.33, n_species_ = 54; queen number pMCMC = 0.43, CI = -0.09–0.05, n_species_ = 51). We found no correlation between worker reproductive potential and queen-worker size dimorphism (BPMM: pM-CMC = 0.19, CI = -0.06–0.31, n_species_ = 32). The significant correlations between queen mating frequency, colony size and reproductive dimorphism highlight the need to carry out analyses that can test the most likely causal explanation.

### Correlations between potential predictors

We found that colony size and effective queen mating frequency are correlated with each other, but that neither colony size nor mating frequency are correlated with queen number (colony size and mating frequency log_10_ MF pMCMC = 0.02, CIs = 0.19–1.96, n_species_ = 57; colony size and queen number log_10_ colony size pMCMC = 0.81, CI = -0.08–0.06, n_species_ = 91; queen number and mating frequency queen number pMCMC = 0.19, CI = -0.12–0.64, n_species_ = 57).

### Phylogenetic path analysis

**Figure 6:**
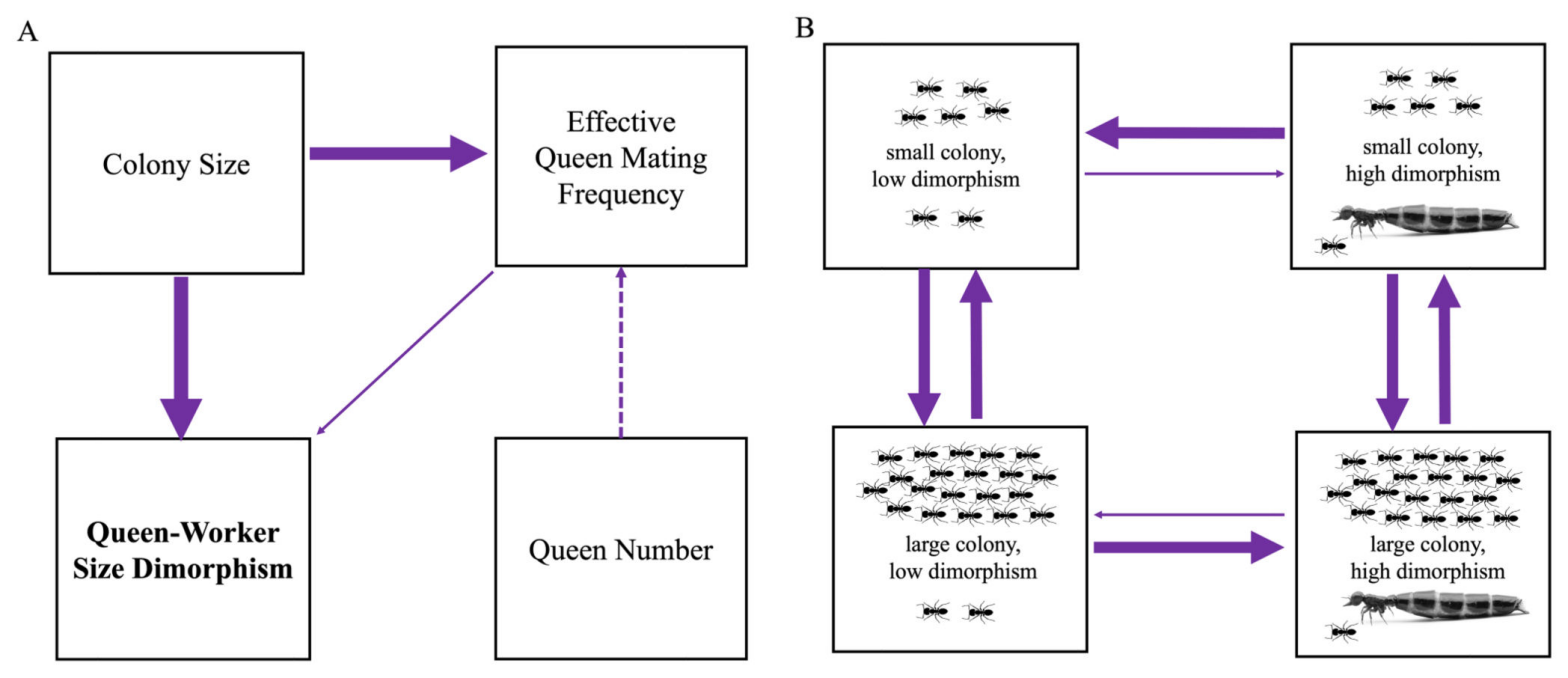
The best-supported causal models identified by phylogenetic path analysis and correlated evolution tests. (A) Path analysis. Larger colony sizes appear to have facilitated the increase of queen-worker size dimorphism. There is some evidence for a weak effect of queen mating frequency on size dimorphism. Here we display the results of one MCC consensus tree but repeated the analysis for three more which can be found in the Supplementary Data. Arrows indicate the direction of the relationship between variables, with heavier lines indicating larger path coefficients. A dashed line indicates a negative relationship, while unbroken lines indicate positive relationships. (B) Correlated evolution. The co-evolution of queen-worker size dimorphism and colony size: larger colony sizes appear to have facilitated the increase of queen-worker size dimorphism. Arrows are weighted by mean transition rates, with heavier arrows indicating higher means.

We found strong support for the size-complexity hypothesis, with larger colony sizes favouring the evolution of increased queen-worker size dimorphism (Figure 6A, n _species_ = 47). This result was consistent across phylogenetic trees and robust to the inclusion of supercolonial species (Supplementary Data Tables 7 & 8). We also found a consistent but weak positive effect of queen mating frequency on size dimorphism.

### Correlated evolution

We again found support for the size-complexity hypothesis, with larger colony sizes favouring the evolution of increased queen-worker size dimorphism (Figure 6B, n _species_ = 45). The strength of the correlation between colony size and queen-worker size dimorphism was positive or strong depending on whether we used the median or the mean size dimorphism threshold for grouping species as having “large” or “small” size dimorphism (BF median threshold = 3.5 or BF mean threshold = 6.8, Supplementary Data 1). This analysis required both colony size and queen-worker size dimorphism to be modelled as binary variables—we used the median to divide our data sets into binary but also repeated size with the mean to test the robustness of our conclusions (see methods). This analysis ran across all 400 phylogenetic trees.

We found that ants rarely evolve greater queen-worker size dimorphism in small colonies, but more often do in large colonies. We also found that ants rarely lose high dimorphism in larger colonies but often do in small colonies. In contrast, we did not find support for a causal relationship between queen mating frequency and size dimorphism (BF = −1.9).

### Ancestral state reconstruction of queen-worker size dimorphism

**Figure 7:**
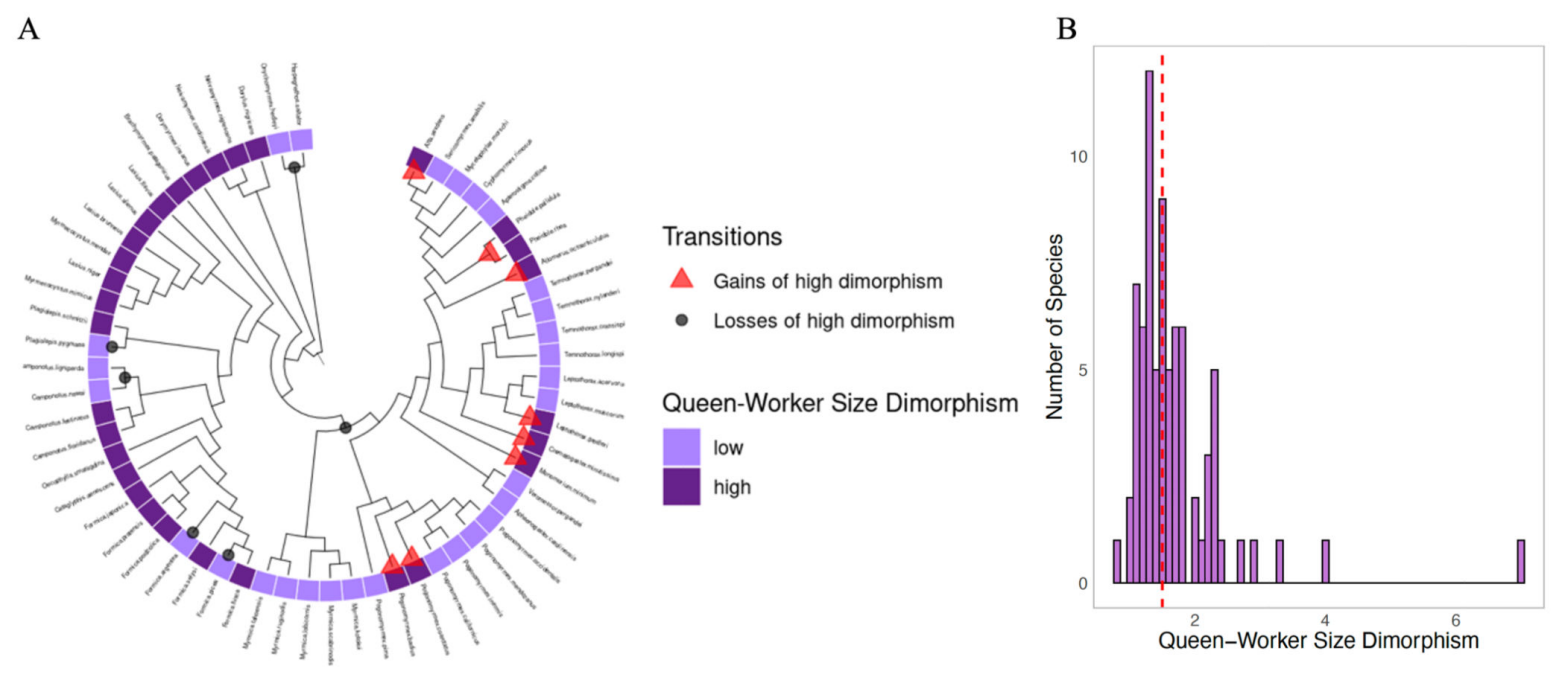
Queen-worker size dimorphism. (A) Ancestral state reconstruction of queen-worker size dimorphism. We reconstructed gains and losses of high size dimorphism using the equal rates model with one rate category. Plot shows transitions of only one tree, but we repeated across 400 possible phylogenies to calculate mean transition numbers (n species = 59). (B) Distribution of queen-worker head size dimorphism. We calculated size dimorphism by mean queen head width divided by minimum worker head width (n species = 76). Larger values indicate greater dimorphism, with queens relatively larger than workers. The median (1.50) is shown by a red dashed line.

When looking at the origins of queen-worker size dimorphism, we estimated a mean of 9 gains of high dimorphism, and 8 losses (Figure 7). The evolution of increased queen-worker size dimorphism was associated with larger colony sizes, but this difference was not significant (Supplementary Figure S2). The most likely root state was high queen-worker size dimorphism (probability low = 0.46, CIs = 0.39–0.50, probability high = 0.54, CIs = 0.50–0.60, n_species_ = 59, “low” if dimorphism ≤ median of dataset, “high” if > median; Supplementary Figure S9).

## Discussion

We found support for the hypothesis that increased colony size favoured greater division of labour between queens and workers in ants, examining both the loss of reproductive capacity in workers and queen-worker size dimorphism (Figures 5 & 6). Our analyses suggest that the “ancestral ant worker” had full reproductive potential, with the ability to mate and produce either male or female offspring, though would not produce offspring in queenright colonies. The loss of reproductive potential appears to have then generally followed a sequential step-by-step process via a reduced ability to produce both males and females, then the loss of the ability to mate (male production only), and finally complete sterility (Figure 3A). Overall, the evolution of complete sterility has occurred around 17 times in ants, with 42% of examined species having completely sterile workers (Figure 3B). Analogously, the evolution of higher queen-worker size dimorphism, where queens can be up to 300 times size of workers, has occurred around 9 times in ants, and the common ancestor was more likely to have greater than average queen-worker size dimorphism.

### The ancestral ant and loss of reproductive potential

Our analyses suggest that the most recent common ancestor of ants likely had workers with full reproductive potential, consistent with recent findings (Meurville et al., 2025). However, we also examined realised reproduction and found that these workers most likely produced no offspring in queen-right colonies, despite their reproductive potential.

There was substantial uncertainty around our root state estimate of reproductive potential, indicating that further data or alternative modelling approaches with additional Hymenopteran species may be needed to fully resolve that state. The closest relatives of the ant family are the Scolioidea and Apoidea (Peters et al., 2017). All Scolioid wasps are solitary, and the most basally divergent lineage of Apoidea is Ampulicidae, which is also solitary (Melo, 2021). By examining realised reproduction as well as reproductive potential, we show that although the ability to reproduce was lost gradually, workers stopped reproducing earlier, and were already effectively sterile (Boomsma, 2022; Linksvayer & Johnson, 2019).

### How was reproductive capacity then lost in ants?

We found strongest support for a model of evolution where the loss of worker reproductive capacity usually occurs in a sequential manner: first reduced capacity for sexual reproduction, then the production of males only and then complete sterility (Figure 3). Ancestral state reconstruction suggested that workers have lost the ability to reproduce sexually 16 times (gained 5 times), and that complete sterility has evolved 17 times (lost 20 times) (Figure 4). Both phylogenetic path analysis and correlated evolution analyses supported the hypothesis that larger colony sizes favoured this loss of worker reproductive potential (Figure 4). The path analysis further suggested that this relationship was bidirectional, with the loss of worker reproductive potential having also favoured larger colony sizes (Figure 4A).

### Colony size and queen-worker size dimorphism

Both phylogenetic path analysis and correlated evolution analyses supported the hypothesis that larger colony sizes had favoured increased queen-worker size dimorphism (Figure 6). Our ancestral state reconstruction suggested that higher queen-worker size dimorphism has evolved 9 times and been lost 8 times (Figure 7).

### The size-complexity hypothesis

There is now support for the hypothesis that larger colony sizes have favoured an increase in both reproductive and non-reproductive division of labour in ants. Examining reproductive division of labour, larger colony sizes appear to have favoured both a reduced reproductive potential (Figure 5) and greater queen-worker size dimorphism (Figure 6). Examining non-reproductive division of labour, we have previously found support for the hypothesis that larger colony sizes favoured more worker castes (Bell-Roberts et al., 2024).

There are several potential hypotheses for why larger colony sizes would select for increased division of labour. In larger colonies, there is a lower chance for each worker to replace the queen if she dies, which could reduce the potential cost of becoming specialised to perform helping functions (Bourke, 1999; Bourke, 2011). Larger colonies may require a greater number of tasks, favouring increased specialisation (Bourke, 2011; Ferguson-Gow et al., 2014). In larger colonies there is a reduced chance of stochastically having a lower than optimal number of individuals performing a task (Cooper et al., 2022; Liu et al., 2021). Larger colonies may be more likely to be associated with non-predatory diets that allow workers greater control of the allocation of resources to offspring (Matte & LeBoeuf, 2025). A combination of comparative and experimental methodologies may be required to test these different hypotheses.

### Queen number and reproductive division of labour

We found weak support for an influence of queen number on size dimorphism and worker reproductive potential. Overall, the relationship between queen number and reproductive division of labour was inconclusive, with different methods producing conflicting results. Phylogenetic path analysis indicated that higher queen number favours the loss of worker sexual reproduction, but that it is not important for overall reduced reproductive potential. Ancestral state reconstructions revealed that differences in queen number were not significant but were associated with a general trend towards fewer queens preceding the loss of worker sex (Supplementary Figure S3E). This result may reflect the increased uncertainty associated with this method which uses all 400 posterior trees, compared to averaged trees used in path analysis and the subset of the 400 used for tests of correlated evolution. In contrast, the tests for correlated evolution identified co-evolution between worker sex and colony size, but not between worker sex and queen number, or between complete worker sterility and either colony size or queen number (Figure 4B).

### Comparison with other studies

Vizueta et al. (2025) performed a comparative analysis of 163 ant genomes and found a positive correlation between larger colony sizes, queen-worker dimorphism, and worker sterility, but they did not test the causal relationship between this correlation.

Matte & LeBoeuf (2025) attempted to test causality but could not determine whether larger colony sizes favoured increased size dimorphism or the reverse. In contrast, we find strong evidence that the evolution of larger colony sizes facilitated greater reproductive division of labour—both queen-worker dimorphism and loss of worker reproductive potential. Our conclusions about causality were robust to three different methods of phylogenetic analysis: path analysis, transition rate analysis, and ancestral state reconstruction. There are several differences in the analytical methods that could potentially explain the difference between studies. These include: (a) different datasets—although we expect broad similarity as they incorporated data from the GAGA project which we had contributed to and cross-checked with (Bell-Roberts et al., 2024; Vizueta et al., 2025); (b) different variables—Matte & LeBouef also included larval passiveness, adult diet, and worker polymorphism, whereas we included queen number and queen mating frequency; (c) different analysis methods—Matte & LeBouef used only phylogenetic path analysis whereas we used multiple methods to test robustness; (d) differences in number of phylogenetic trees used—we used 400 for all analyses except for path analysis, where we used four maximum clade credibility trees, whereas Matte & LeBouef (2025) used 200 posterior trees for path analysis using Multitree phylogenetic path analysis; (e) differences in the possible evolutionary models considered.

Meurville et al. (2025) examined the relationships between worker reproductive autonomy, stingers, colony size, trophallaxis, and diet. They found that loss of worker reproductive autonomy promoted the evolution of trophallaxis, which in turn contributed to enlarged colony sizes. However, they did not test alternative causal models for the relationship between worker reproduction and colony size, testing only one causal direction and keeping it consistent while varying the paths of influence between their other variables. They also examined worker reproduction as a binary presence or absence of reproductive gamergate workers, whereas we measured reproductive potential as four categories (Figure 2). Meurville et al. (2025) reconstructed the reproductive potential of the ancestral ant, finding that the ancestor most likely had full reproductive potential—a result that we confirm with our own analyses here (Figure 4). We expanded on this by examining realised reproduction and found that although ancestral workers had full reproductive potential, they most likely did not reproduce in queenright colonies, and so were already effectively sterile (Boomsma, 2022; Linksvayer & Johnson, 2019).

### Losses and gains of organisational complexity—other systems

Our results demonstrated that organisational complexity may be more changeable than expected in ants. We found that ant workers have gained and lost reproductive potential many times over, with changes occurring both as increases and decreases in complexity (complexity defined here as mutual dependence and specialisation, e.g., 17 gains of worker sterility, 20 losses). This illustrates that evolution does not occur in a simple linear progression from simpler to more complex life forms (Boomsma, 2022; Linksvayer & Johnson, 2019). Losses of previously gained organisational complexity have been found in other insects, such as the loss of a soldier caste in termites, aphids, and thrips (Revely et al., 2024; Chapman et al., 2008; Stern & Foster, 1996). Another example is provided by the emergence of transmissible cancers, where cells formerly part of a cooperative multicellular organisms break away and proliferate independently, sometimes even infecting other species as hosts (Garcia-Souto et al., 2022). This is analogous to the loss of colonial living in lineages that have become social parasites, dependent on the social structure of another species but lacking their own caste system (Chapuisat, 2023).

## Conclusions

We have provided clear support for how colony size can influence the evolution of reproductive division of labour in ants, but there are at least three open questions: First, in ants, what came first—reproductive (sterility and queen-worker dimorphism) or non-reproductive (worker castes) division of labour (Molet et al., 2012). Second, does colony (or group) size play a similar role in other insect lineages, including those that have not undergone a major transition to superorganismality? Third, does the number of cells have an analogous causal influence on division of labour in multicellular groups?

## Supplementary Material

Supplementary material is available online at Evolution.

Supplementary Material

## Data Availability

Analysis scripts are available on GitHub: https://github.com/jfturner6/ant_queen_worker_dimorphism
