# Supplementary material for: Larger colony sizes favour greater division of labour between queens and workers in ants
Source: Evolution. Author manuscript; Available in PMC 2026 Mar 10. (PMC7618842; doi:10.1093/evolut/qpaf250)
Supplement: Supplementary Material [file EMS212756-supplement-Supplementary_Material.zip › Supplementary Material.pdf]

Supplementary Material

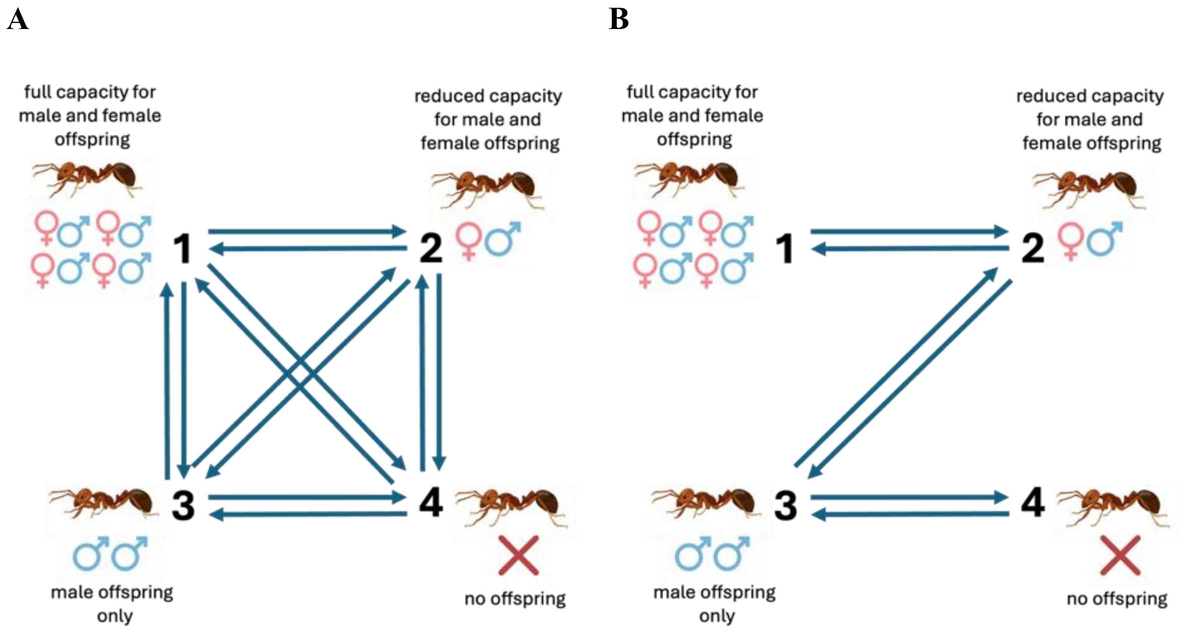

Figure S1. Modelling evolutionary transitions in worker reproductive potential. (A) The 'free' model. All transitions between states are equally possible (B) The 'sequential' model. Non-sequential transitions are restricted to zero.

Ancestral State Reconstructions

1. <u>Colony size and queen-worker size dimorphism</u>

We found no evidence that ancestral colony sizes were larger in lineages that transitioned to a state of high queen-worker size dimorphism when compared to lineages that did not have this transition (high dimorphism: posterior mode = 2435, HPD = 135 – 24054; low dimorphism retained: posterior mode = 423, HPD = 33 – 4287, $p_{\text{high vs low}} = 0.84$; Figure SI 1A). When looking at colony size before and after transitions to high dimorphism, we found no evidence that colony size may increase following transitions to high dimorphism (before high dimorphism: posterior mode = 2352, HPD = 134 – 24334; after high dimorphism: posterior mode = 5257, HPD = 138 – 147967, $p_{\text{after vs before}} = 0.67$; Figure SI 1B).

2. <u>Queen mating frequency and queen-worker size dimorphism</u>

We compared queen mating frequency in lineages that transitioned to high queen-worker size dimorphism with lineages where they remained in a state of low size dimorphism and found that there was no significant difference in queen mating (high dimorphism: posterior mode = 3.49, HPD = -2.29 – 10.72; low dimorphism: posterior mode = 3.83, HPD = -3.06 – 11.38, p transition to high > low = 0.50; Figure SI 1 C). We also compared queen mating frequency before and after transitions to high dimorphism within the lineages that had this transition and again found no significant difference (before: posterior mode = 3.58, HPD = -2.29 – 10.37; after: posterior mode = 4, HPD = 1.22 – 7.31, p after > before = 0.55; Figure SI 1 D).

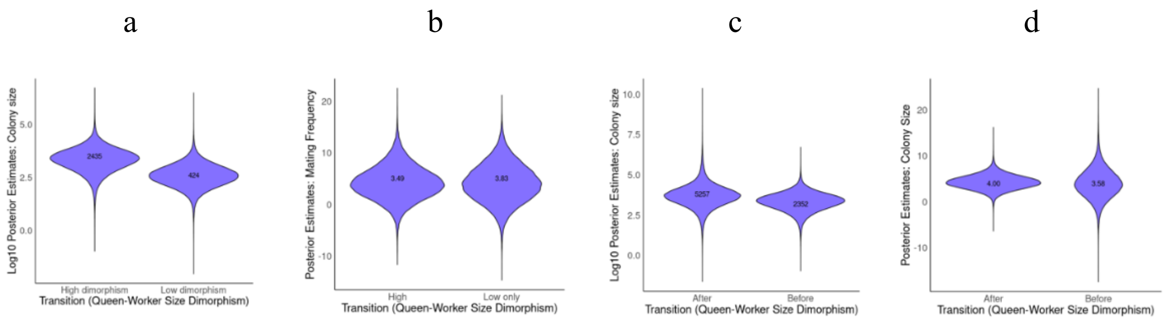

Figure S2. Ancestral state reconstructions of colony traits and queen-worker size dimorphism. (A) Colony size in lineages that transitioned to greater size dimorphism compare dot lineages that retained smaller size dimorphism, **(B)** Colony size before and after transitions to greater size dimorphism, **(C)** Queen mating frequency in lineages that transitioned to greater queen-worker size dimorphism compared to lineages that retained smaller size dimorphism, (D) Queen mating frequency before and after transitions to greater queen-worker size dimorphism.

3. Colony size and worker sex

We found no evidence that colony sizes may be larger in lineages that lost worker sex when compared to those that retained sex (sex lost: posterior mode = 708, HPD = 25 – 19,957; sex retained: posterior mode = 252, HPD = 9 – 5650, $p_{\text{lost vs retained}} = 0.14$; Figure SI 2A). We also compared colony size before and after losses of sex ('sex.both' vs no.sex.only.no.sex') to test the alternative hypothesis and see if colony sizes increase following losses of sex, but again there was no significant difference (before loss: posterior mode = 708, CI = 25 – 19957; after loss: posterior mode= 1158, CI = 72 – 233690, $p_{\text{after vs before}} = 0.28$; Figure SI 2B).

4. Colony size and worker sterility

Colony sizes may be larger when there are transitions to worker sterility than in lineages that retained some reproductive potential, but this difference was not significant (transitions to sterility: posterior mode = 2069, HPD = 57 – 90556; non-sterile: posterior mode = 596, HPD = 32 – 10325, $p_{\text{sterility vs no sterility}} = 0.87$; Figure SI 2C). When looking at colony size before and after transitions to sterility, we found no evidence that colony sizes are larger after transitions to sterility than before transitions (before transitions to sterility: posterior mode = 2069, HPD = 57 – 90556; after transitions to sterility: posterior mode = 1540, HPD = 78 – 50707, $p_{\text{before vs after sterility}} = 0.48$; Figure SI 2D).

5. Queen number and worker sex

We found no evidence that there may be fewer queens per colony in lineages where workers lost their sexual capacity when compared to lineages where sexual capacity was retained (sex lost: posterior mode = 1.78, HPD = 0.23 – 10.70; sex retained: posterior mode = 4.56, HPD = 1.37 – 16.36, $p_{\text{sex retained > sex lost}} = 0.18$; Figure SI 2E). We then compared queen numbers in lineages that lost worker sexual capacity before and after this loss, and found no significant

difference, although lineages that lost sex had a much wider confidence interval than those that only had no sex (loss of sex: posterior mode = 1.78, HPD = 0.23 – 10.70; only no sex (after loss): posterior mode = 1.56, HPD = 1.15 – 2.26, $p_{\text{loss of sex > only no sex}}$ = 0.49; Figure SI 2F).

6. Queen number and sterility

We found no evidence that there were fewer queens in lineages that had transitions to worker sterility when compared to lineages that remained non-sterile (transitions to sterility: posterior mode = 1.59, CI = 0.70 – 5.35; remained non-sterile: posterior mode = 1.83, CI = 1.29 – 2.75, $p_{\text{non-sterile vs sterile}}$ = 0.45; Figure SI 2G). When comparing queen number before vs after transitions to worker sterility to see if queen number may decrease as a result of worker sterility, we also found no significant difference (before sterility: posterior mode = 1.59, CI = 0.70 – 5.35; after sterility: posterior mode = 1.54, CI = 0.79 – 3.36, $p_{\text{before vs after}}$ = 0.43; Figure SI 2H).

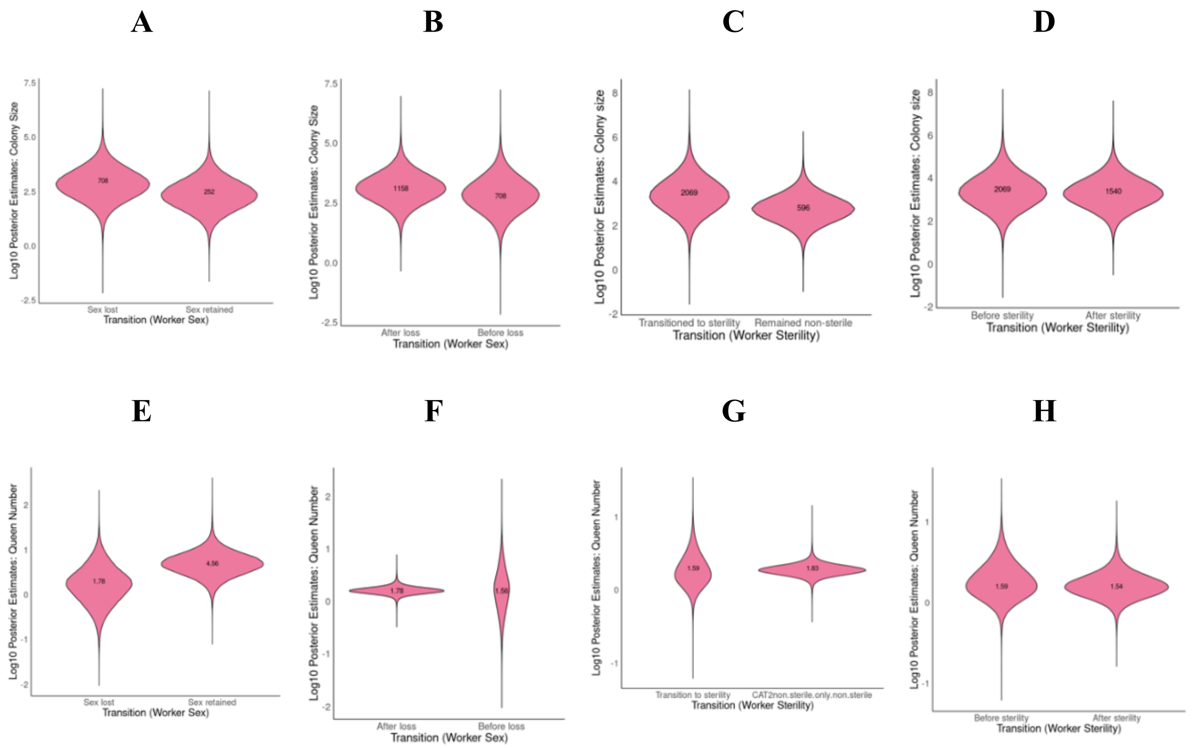

Figure S3. Ancestral state reconstructions of worker reproductive potential. (A - H) Reconstructions of ancestral colony size and queen number at points of gains or losses of worker reproductive potential.

Phylogenetic Path Analysis

7. Worker sex analysed as a binary variable

We also analysed reproductive potential as binary sex/no sex (categories 1 & 2 vs. 3 & 3) to see if there are distinct factors influencing the loss of worker sex that would not be important in overall loss of worker reproductive potential. We found consistent evidence that loss of sex favours increased colony size, with the opposite causal direction also possible, although this direct was not as strong or consistent (Supplementary Material Figure 4). We found that this time, queen number was important: More queens per colony favours the loss of worker sex. All models showed this relationship, remaining consistent across subsets and phylogenetic trees (Figure 7B).

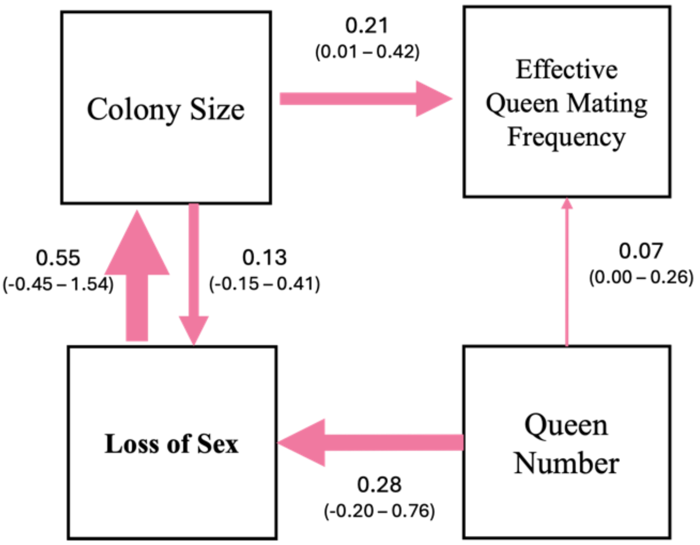

Figure S4. Queen number favours the loss of worker sex, and loss of sex favours larger colony sizes, with causality potentially existing in the opposite causal direction. Worker reproductive potential was analysed as binary sex/no sex (full or reproduced sexual

capacity vs. males only or no offspring; categories 1 & 2 vs. 3 & 4; Figure 2). All other variables are continuous. Here we display the results of our highest confidence data ($n_{species}$ = 44) and the FBD Crown MCC consensus tree. Values represent standardized path coefficients with 95% confidence intervals. Arrows indicate the direction of the relationship between variables, with heavier lines indicating larger coefficients.

8. Alternative causal models tested: Queen-worker size dimorphism.

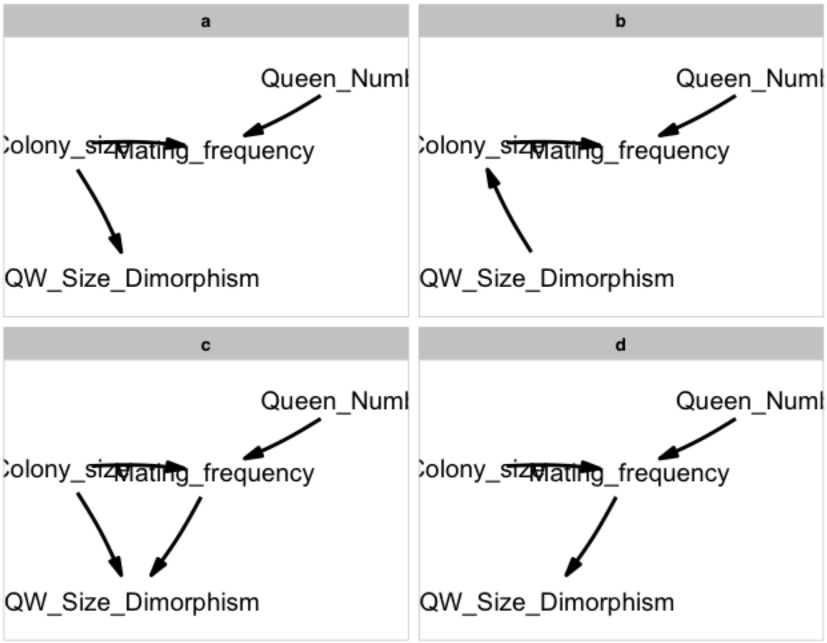

Figure S5. The alternative causal models we tested using phylogenetic path analysis, based on the results of our correlational tests. We alternate the paths of influence to test whether **(A)** colony size is responsible for increases in size dimorphism, **(B)** size dimorphism is responsible for increases in colony size, **(C)** colony size and queen mating frequency are both responsible for increases in size dimorphism, or **(D)** only queen mating frequency is responsible for increases in size dimorphism.

9. <u>Alternative causal models tested: Worker reproductive potential.</u>

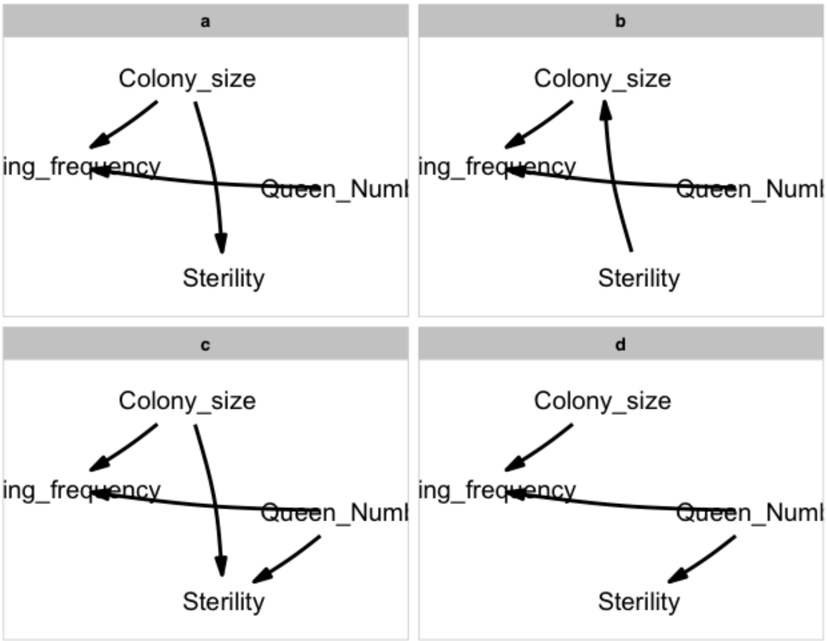

Figure S6. The alternative causal models we tested using phylogenetic path analysis, based on the results of our correlational tests. We alternate the paths of influence to test whether **(A)** colony size is responsible for reduced worker reproductive potential (sterility), **(B)** worker sterility is responsible for increases in colony size, **(C)** colony size and queen number are both driving worker sterility, or **(D)** only queen number is driving worker sterility.

Root state estimates

10. Root state of worker reproductive potential.

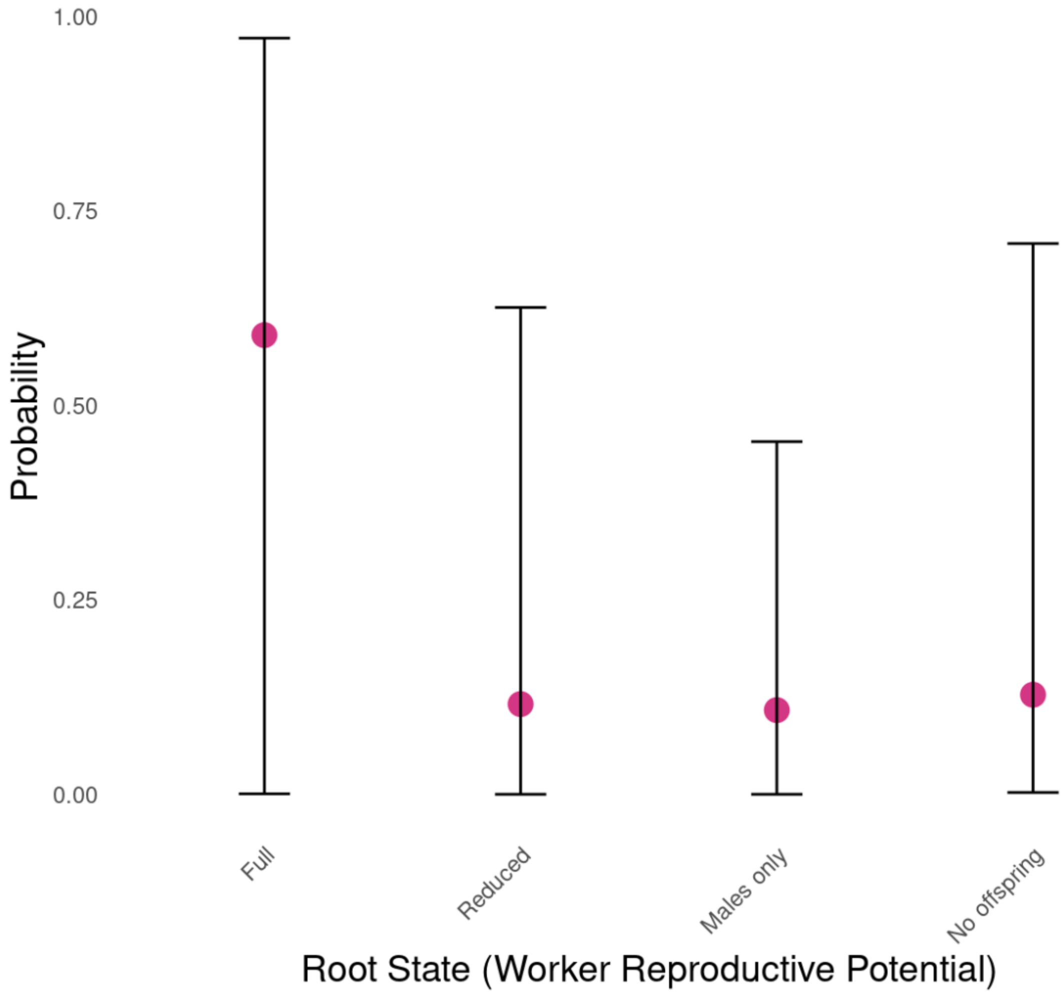

Figure S7. The ancestral ant worker most likely had full reproductive potential, though all confidence intervals contain values close to zero, reflecting substantial uncertainty.

11. Root state of worker realised reproduction.

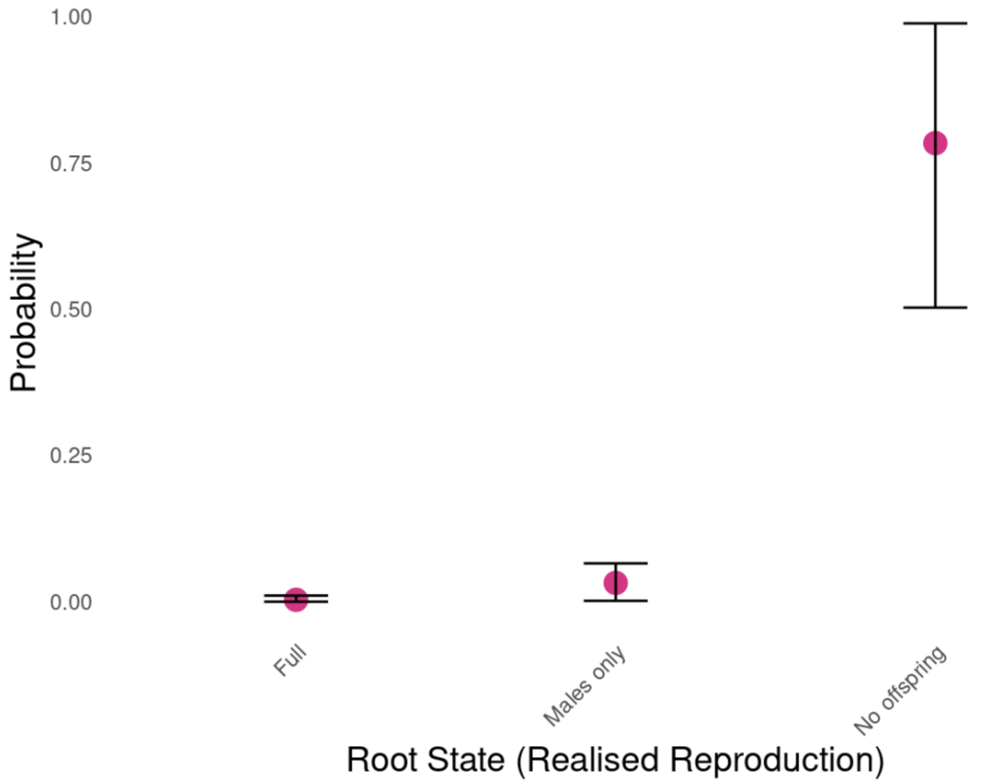

Figure S8. It is very likely that ancestral workers were effectively sterile, producing no offspring in the presence of a queen or gamergate queen.

12. Root state of queen-worker size dimorphism

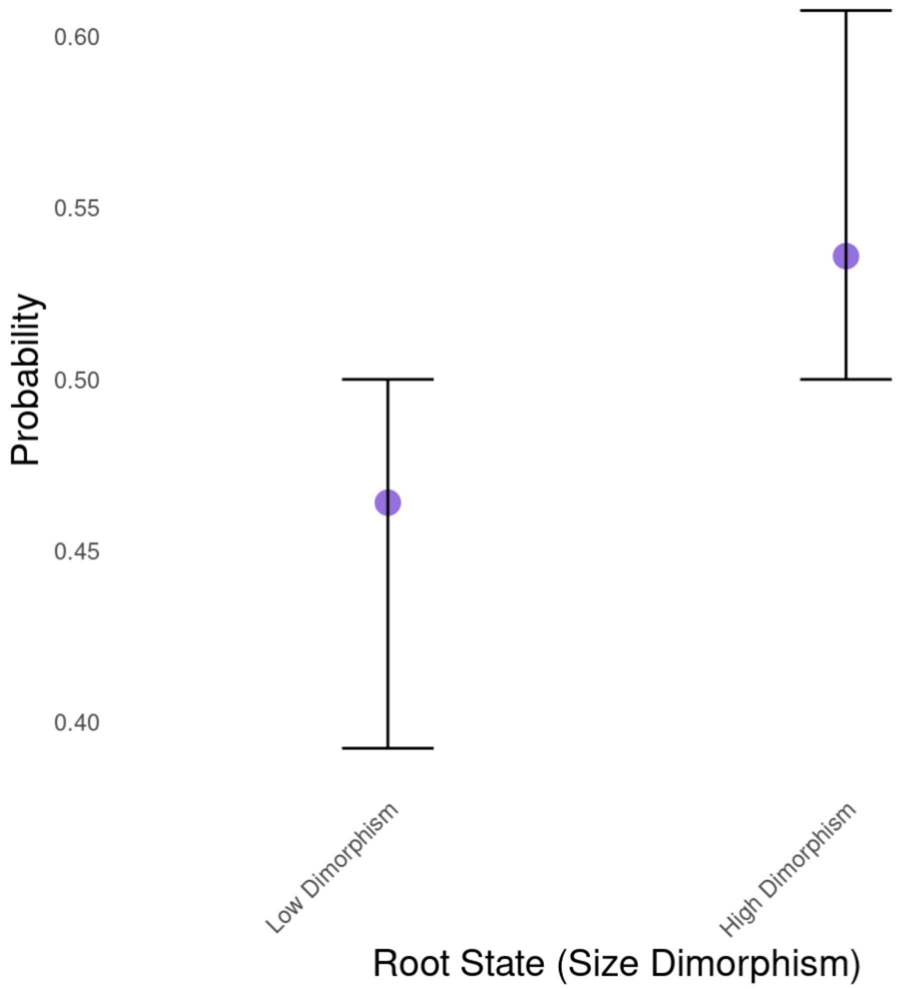

Figure S9. The ancestral ant most likely had high queen-worker size dimorphism.
